# *ESR2* Drives Mesenchymal-to-Epithelial Transition in Triple-Negative Breast Cancer and Tumorigenesis *In Vivo*

**DOI:** 10.3389/fonc.2022.917633

**Published:** 2022-06-03

**Authors:** Zoi Piperigkou, Anastasios Koutsandreas, Marco Franchi, Vasiliki Zolota, Dimitrios Kletsas, Alberto Passi, Nikos K. Karamanos

**Affiliations:** ^1^Biochemistry, Biochemical Analysis and Matrix Pathobiology Research Group, Laboratory of Biochemistry, Department of Chemistry, University of Patras, Patras, Greece; ^2^Foundation for Research and Technology-Hellas (FORTH)/Institute of Chemical Engineering Sciences (ICE-HT), Patras, Greece; ^3^Department for Life Quality Study, University of Bologna, Rimini, Italy; ^4^Department of Pathology, School of Medicine, University of Patras, Patras, Greece; ^5^Laboratory of Cell Proliferation and Ageing, Institute of Biology, National Centre for Scientific Research (N.C.S.R). “Demokritos”, Athens, Greece; ^6^Department of Medicine and Surgery, University of Insubria, Varese, Italy

**Keywords:** breast cancer, ESR2, estrogen receptor beta, tumorigenesis, extracellular matrix

## Abstract

Estrogen receptors (ERs) have pivotal roles in the development and progression of triple-negative breast cancer (TNBC). Interactions among cancer cells and tumor microenvironment are orchestrated by the extracellular matrix that is rapidly emerging as prominent contributor of fundamental processes of breast cancer progression. Early studies have correlated ERβ expression in tumor sites with a more aggressive clinical outcome, however ERβ exact role in the progression of TNBC remains to be elucidated. Herein, we introduce the functional role of ERβ suppression following isolation of monoclonal cell populations of MDA-MB-231 breast cancer cells transfected with shRNA against human *ESR2* that permanently resulted in 90% reduction of ERβ mRNA and protein levels. Further, we demonstrate that clone selection results in strongly reduced levels of the aggressive functional properties of MDA-MB-231 cells, by transforming their morphological characteristics, eliminating the mesenchymal-like traits of triple-negative breast cancer cells. Monoclonal populations of shERβ MDA-MB-231 cells undergo universal matrix reorganization and pass on a mesenchymal-to-epithelial transition state. These striking changes are encompassed by the total prevention of tumorigenesis *in vivo* following ERβ maximum suppression and isolation of monoclonal cell populations in TNBC cells. We propose that these novel findings highlight the promising role of ERβ targeting in future pharmaceutical approaches for managing the metastatic dynamics of TNBC breast cancer.

## Introduction

Human cancers arise from multistep processes that make their way from normalcy to the acquisition of particular hallmark traits during complex tumorigenic signaling cascades (1). Breast cancer is characterized by a great heterogeneity in its molecular subtypes, therefore important breakthroughs reducing relapse and providing higher quality years of life may be accomplished in treatment approaches.

Estrogens as master regulators of breast cancer susceptibility, mediate their effects in target tissues through two estrogen receptors (ERs), ERα and ERβ. ERs and their variants exert distinct functions following activation in response to ligand binding and trigger genomic and non-genomic signaling cascades (2). Many lines of evidence suggest that in breast cancer, ER-evoked signaling is closely connected to extracellular matrix (ECM) remodeling that stimulates cancer progression, metastasis and drug resistance (3). The role of tumor ECM has long been recognized as a dynamic 3D structural and functional network of biomolecules that dynamically interact to reinforce cancer cell properties (4). Major matrix components of this functional bioscaffold consist, among other constituents, of collagen, proteoglycans (PGs), glycosaminoglycans (GAGs), adhesive glycoproteins, fibrils and degrading enzymes, that actively communicate to orchestrate ECM renewal, cell morphology and functional properties of cancer cells (5–7). The integrity of ECM composition is critical for normal tissue homeostasis, since altered expression of ECM macromolecules in the tumor microenvironment (TME) affects cancer cell survival, growth, migration, and invasion to adjacent tissues (8, 9). The triple-negative breast carcinoma (TNBC), accounting for up to 20% of breast carcinomas, is the aggressive molecular subtype of breast cancer, characterized by the absence of ERα, progesterone receptor (PgR), and HER2. Population-based studies show that TNBC is more common to younger age groups of premenopausal African American and Hispanic women compared to Caucasian American women (10).

There is increasing evidence that the second ER isoform, ERβ, which is localized in myoepithelial cells as well as in the surrounding stroma and endothelial cells, is highly expressed and correlated to worse survival rates in TNBC patients (3, 11). The discovery of ERβ in 1996 as the second nuclear receptor for steroid/thyroid hormones (i.e., 17β-estradiol, E2), after ERα, reserved a new era in the diagnosis, survival estimation and therapeutic targeting of breast cancer (12). While less well established, ERβ dynamically communicates with major matrix components, including PGs and epidermal growth factor receptor (EGFR) to stimulate cancer cell behavior, epithelial-to-mesenchymal transition (EMT) and stem-like characteristics (13–15).

EMT is an evolutionarily conserved developmental program in which cells gradually unbend tight cell junctions due to the decreased expression of epithelial proteins (i.e., E-cadherin) and gain mesenchymal traits through epigenetic alterations, reorganized cytoskeleton and the expression of mesenchymal matrix markers, such as fibronectin and vimentin (16). This process is the driving force of cancer cells to increased motility and initiation of metastasis, through matrix remodeling and the activation of signaling cascades (i.e., Notch, TGFβ) and a possible mechanistic basis for anticancer drug resistance (17, 18).

The daunting consequences of TNBC arise from matrix extensive reorganization and EMT activation builds the aggressive cancer cell behavior that establishes the initiation of metastasis. These functional capabilities acquired by TNBC cells may be the motive power to advance modern TNBC targeting approaches for the diagnosis and personalized therapeutic management. Recent work has revealed some encouraging data correlating *ESR2* suppression in TNBC cells with a less aggressive cell phenotype (14, 19, 20). Similar to other receptors for steroid hormones, ERβ, encoded by *ESR2* gene, is expressed as a pool of five alternatively spliced variants; the wild-type, ERβ1 and also ERβ2, ERβ3, ERβ4 and ERβ5, which exist in normal and disease states (2). In breast cancer tissues, the most common ERβ variants are ERβ1, ERβ4 and ERβ5, which may be dimerized in order to boost signal transduction processes (21, 22). This explains that shERβ MDA-MB-231 cells demonstrated phenotypic heterogeneity that was reflected in deviant phenotype and *ESR2* mRNA levels; from 70% to 80% inconstant decrease in *ESR2* levels and intrigued us to achieve higher ERβ gene and protein suppression rates to exclude the impact of ERβ variants on heterogenous profile of ERβ-suppressed cells.

Herein, we report for the first time that the isolation of monoclonal cell populations, characterized by ERβ knockdown, led to cultures with a constant epithelial-like behavior. Certainly, such clues force further investigation ERβ as a power player of the tumor microenvironment of TNBC cells. This prompted us to further elucidate the functional effects of ERβ knockdown as compared to parental breast cancer cell lines with distinct ER status, as well as the *in vivo* tumorigenic effects of monoclonal shERβ MDA-MB-231 cell populations. A detailed molecular understanding of ERβ functions is critical in identifying its promising role in TNBC targeting.

## Materials and Methods

### Cell Cultures, Transfections and Selection of Monoclonal Cell Populations

MDA-MB-231 (high metastatic potential; ERβ-positive) and MCF-7 (low metastatic potential; ERα-positive) breast cancer cell lines were obtained from the American Type Culture Collection (ATCC). MDA-MB-231 cells were routinely cultured in a humidified 95% air/5% CO_2_ incubator at 37°C in complete medium [Dulbecco’s Modified Eagle’s Medium (DMEM) supplemented with 10% fetal bovine serum (FBS), 1.0 mM sodium pyruvate, 2 mM L-glutamine and a cocktail of antimicrobial agents (100 IU/ml penicillin, 100 μg/ml streptomycin, 10 μg/ml gentamicin sulfate and 2.5 μg/ml amphotericin B)]. Cells were harvested by trypsinization with 0.05% (w/v) trypsin in PBS containing 0.02% (w/v) Na_2_EDTA. Transfections of MDA-MB-231 cells with shRNA against human *ESR2* or non-targeting shRNA control were performed as previously described by Piperigkou et al. (14), and ERβ suppression was monitored with real-time PCR analysis and western blot analysis.

Since the cultures of stably transfected shERβ MDA-MB-231 cells were heterogenous in respect of *ESR2* levels, the isolation of monoclonal cell populations (clone selection) was performed to identify single clones with the highest *ESR2* suppression levels, as follows. Briefly, 10 cells/ml of shERβ MDA-MB-231 cells were seeded in a 96-well culture plate adding 100 μl per well (i.e., 1 cell per well), the number of cells per well after 24 hours was assessed and the wells with only 1 cell were noted. The monoclonal population has been expanded and passaged to 6-well plates. A portion of cells was examined for the levels of ESR2 suppression and expression stability and the cultures were further expanded. Finally, suppressed clones (~90% ESR2/ERβ suppression) were freezed down and named “clone shERβ MDA-MB-231” cells). Notably, during the freeze-thaw cycle of clone shERβ MDA-MB-231 cells the suppression rates of *ESR2* and ERβ are totally stable, as confirmed by real-time PCR and western blot analysis, respectively.

### Chemicals and Reagents

DMEM, FBS, L-glutamine, penicillin, streptomycin were all obtained from Biosera (Nuaillé, France). All other chemicals used were of the best commercially available grade.

### RNA Isolation, Reverse Transcription and Real-Time qPCR Analysis

Total cellular RNA was isolated using NucleoSpin RNA II Kit (Macherey-Nagel, Duren, Germany). The amount of isolated RNA was quantified by measuring its absorbance at 260 nm. Total RNA was reverse transcribed using the PrimeScript 1^st^ strand cDNA synthesis kit perfect real time (Takara Bio Inc., Japan). Real-time qPCR analysis was conducted in 20 μl reaction mixture, according to manufacturer’s instructions (KAPA Taq ReadyMix DNA Polymerase, KAPA BIOSYSTEMS, Wilmington, Massachusetts). The amplification was performed utilizing Rotor Gene Q (Qiagen, USA). All reactions were performed in triplicates and a standard curve was always included for each pair of primers for assay validation. In addition, a melting curve analysis was always performed for detecting the SYBR Green-based objective amplicon. To provide quantification, the point of product accumulation in the early logarithmic phase of the amplification plot was defined by assigning a fluorescence threshold above the background, defined as the threshold cycle (Ct) number. Relative expression of different gene transcripts was calculated by the ΔΔCt method. The Ct value of any gene of interest was normalized to the Ct of the normalizer (GAPDH). Fold changes (arbitrary units) were determined as 2^-ΔΔCt^. Primer sequences of the tested genes are presented in **Supplementary Table 1**. All primers were purchased from Eurofins Genomics (Ebersberg, Germany).

### Tumorigenicity Assay

An equal number (106) of MDA-MB-231 or clone shERβ MDA-MB-231 cells was inoculated in the back of seven 5-weeks-old SCID female mice. Four weeks later, the animals were sacrificed, and the tumors formed following MDA-MB-231 inoculations were removed. The tumor volume was calculated with the Caliper method, using the formula tumor volume = 1/2(length × width^2^) (23). Tumor samples developed from MDA-MB-231 cells were mechanically homogenized in the presence of liquid nitrogen and stored in -80°C. All animal studies were conducted according to the institutional guidelines conforming to international standards and the protocols were approved by the relevant committee of the Veterinary Direction, Greek Ministry of Rural Development and Food (Permission No. 193900).

### Immunohistochemistry

Serial 3μm paraffin sections were cut from tissue blocks, mounted on poly-L-lysine-coated slides and subjected to immunohistochemical staining. Briefly, the sections were initially dried for 24 hours at 60°C, deparaffinized in xylene and dehydrated in gradient alcohol. Antigen retrieval was performed at 600W in a microwave for 20 minutes. Endogenous peroxidase blocking was performed by incubating the slides in a 3% H_2_O_2_ solution for 15 minutes. Sections were then incubated with the following primary antibodies against: ERα, clone 6F11 (Leica Biosystems), Ki-67 IHC MIB-1 (DAKO, mouse monoclonal, 1:50), HER2 (DAKO, Rabbit polyclonal, 1:300), E-cadherin (DAKO, mouse monoclonal, 1:50), vimentin, clone V9 (Leica Biosystems). Dako EnVision polymer (Dako EnVision Mini Flex, Dako Omnis, Angilent Technology Inc., California, USA, GV823) was used for signal detection. Diaminobenzidine (Dako Omnis, GV823) was used as a chromogen and Harris hematoxylin was used for nuclear counterstaining. Positive and negative controls for antibody validation were used according to the manufacturer’s instructions.

### Western Blot Analysis

Cell monolayers were washed with cold PBS and lysed with Lysis Buffer: 25 mM Hepes, pH 7.5, 150 mM NaCl, 5 mM EDTA, 10% (v/v) glycerol, 1% (v/v) Triton X-100, containing protease inhibitor cocktail (#20-201 Chemicon, Millipore, CA) and 0.5 mM sodium orthovanadate (S6508, Sigma-Aldrich, Inc). Samples were reduced with β-mercaptoethanol in Laemmli sample buffer, separated by SDS-PAGE in 12% poly-acrylamide gels and transferred to polyvinylidene difluoride (PVDF) membranes (Macherey Nagel, Germany). The membranes were blocked in 5% (w/v) non-fat dry milk in Tris-buffered saline pH 7.4 containing 0.05% Tween-20 (TBS-T) for 2 hours at room temperature and were then incubated with primary antibodies for 16 hours at 4°C. After three washes in TBS-T, membranes were further incubated with peroxidase-conjugated secondary goat anti-rabbit IgG (A0545, Sigma-Aldrich, Inc) or anti-mouse IgG (A4416, Sigma-Aldrich, Inc) for 90 minutes at room temperature. Detection of the immunoreactive proteins was performed by chemiluminescence horseradish peroxidase substrate Super Signal (Pierce, Thermoscientific), according to the manufacturer’s instructions. Primary antibodies used in immunoblotting include ERβ **(**ab3576, abcam), p-ERK1/2 (9101, Cell Signaling Technology, dilution 1:1000), total ERK1/2 (9102, Cell Signaling Technology, dilution 1:1000) and α-tubulin (T9026, Sigma-Aldrich Inc., dilution 1:7500). ImageJ software has been used for measuring the band density.

### Immunofluorescence and Phase-Contrast Microscopy

For immunofluorescence microscopy, parental and transfected breast cancer cells were seeded on glass coverslips in 24-well plates and grown to confluence. Cells were first washed twice with phosphate-buffered saline (PBS) buffer, fixed in 4% formaldehyde in PBS buffer, washed three times with PBS-Tween buffer, permeabilized with freshly made 0.5% Triton X-100 in PBS, washed three times with PBS-Tween buffer and blocked with 5% BSA in PBS. Slides were stained for E-cadherin, vimentin and F-actin with the following primary antibodies: E-cadherin (ECCD-2, Takara, dilution 1:200) and Alexa-Fluor 568-labeled phalloidin (Invitrogen Corporation, Carlsbad, USA, dilution 1:100. Then the appropriate Alexa Fluor-488 anti-mouse (A-11032, Invitrogen, dilution 1:2000) secondary antibody was used for E-cadherin staining and the coverslips were mounted on microscope slides. Stained slides with the appropriate secondary antibodies alone were used as negative controls. For phase-contrast microscopy, images of live cells growing on the culture dish were collected on an OLYMPUS CKX41 microscope equipped with a CMOS color digital camera (SC30). Cell circularity was monitored using the ImageJ plugin that calculates object circularity using the formula: circularity= 4pi(area/perimeter^2^). This formula was applied to each cell of 10 representative digital images of parental and transfected breast cancer cells. A circularity value of 1.0 indicates a perfect circle. As the value approaches 0.0, it indicates an increasingly elongated polygon.

### Scanning Electron Microscopy

Parental and transfected breast cancer cells seeded in culture flasks for 48 hours, were firstly rinsed with a phosphate buffer solution to prevent cells detachment and then fixed in a Karnovsky’s solution for 20 minutes. Flasks with adhering cells were again rinsed three times with 0.1% cacodylate buffer, post-fixed in 1% OsO_4_ in cacodylate buffer for 20 minutes, dehydrated with increasing concentrations of ethanol, and finally dehydrated with hexamethyldisilazane (Sigma-Aldrich Inc.) for 15 minutes. The specimens were mounted on appropriate stubs, coated with a 5nm palladium gold film (Emitech 550 sputter-coater) to be observed under a SEM (Philips 515, Eindhoven, The Netherlands) operating in secondary-electron mode.

### Cell Viability Assay

Parental and transfected breast cancer cells were seeded in the presence of FBS into 96-well plates at a density of 5,000 cells/well and then the cells were incubated in serum-free culture medium. Premix WST-1 (water-soluble tetrazolium salt) Cell Proliferation Assay System (Takara Bio Inc., Japan) was added after 24 hours at a ratio 1:10. The assay is based on the reduction of WST-1 by viable cells, producing a soluble formazan salt absorbing at 450 nm (reference wavelength at 650 nm).

### Wound Healing Assay

Parental and transfected breast cancer cells were seeded in 12-well cell culture plates at a density of 25,000 cells/well. Breast cancer confluent cell layers were serum starved for 16 hours and then wounded by scratching with a sterile 100 μl pipette tip. Detached cells were removed by washing twice with PBS and fresh culture medium, in the absence of FBS, was added. The wound closure was monitored at 0 and 24 hours using a digital camera connected to a microscope. Wound surface area was quantified by image analysis (Image J software).

### Collagen Invasion Assay

The invasive potential of parental and transfected breast cancer cells was evaluated as previously described (24). In brief, the collagen type I solution with final concentration of 1 mg/ml was prepared by mixing the precooled components: 4 volumes collagen type I (stock concentration 3 mg/ml), 5 volumes of CMF-HBSS, 1 volume of MEM (10x), 1 volume of 0.25 M NaHCO_3_, 2.65 volumes of standard medium and 0.3 volumes of 1M NaOH. The solution was gently mixed and added to one well of 12-well plate, spread homogeneously and let gelify in a humidified atmosphere of 10% CO_2_ at 37°C for at least 1 hour. Cells were serum-starved overnight and then seeded at a density of 6x10^4^ cells/well on top of collagen I type gels and cultured for 24 hours. Digital images were obtained with 10x objective and the evaluation of cell invasion was conducted according to the experimental protocol (24).

### Cell Adhesion Assays

In order to evaluate the adhesion potential of breast cancer cells, the following adhesion protocol was performed, as previously described (25). Briefly, 96-well plate was coated with collagen type I (40 μg/ml) and kept at 4°C. After 12 hours, the solution was removed, and the plate was air-dried; 3% BSA in PBS solution was added in each well, for 30 minutes, to block non-specific adsorption. Then the solution was removed, and the plate was washed with PBS and air-dried. Cells treated for 24 hours prior to the adhesion assay were detached with PBS-EDTA 1x and resuspended in serum-free medium with 0.1% BSA. and seeded at a density of 2x10^4^ cells/well. Cells were incubated for 30 min, to allow adhesion to the surface. Non-adherent cells were removed with serum free medium, and then cells were incubated with medium supplemented with 10% FBS for 2 hours for recovery. Premix WST-1 (water-soluble tetra-zolium salt) Cell Proliferation Assay System (Takara Bio Inc., Göteborg, Sweden) was then added at a ratio 1:10, and the absorbance at 450 nm was measured (reference wavelength at 650 nm).

### Prognostic Power of *ESR2*

Data on the *ESR2* isoform structure, the interactive bodymap, the signature-based statistics for normal/cancer comparison, and Kaplan-Meier overall survival were collected byGEPIA2, the online server for large-scale analysis of cancer-related genomic datasets (26). GEPIA2 is a highly cited resource for analyzing the RNA sequencing expression data of 9,736 tumors and 8,587 normal samples from the TCGA and the GTEx databases, using a standard processing pipeline applying the bioinformatics tools CIBERSORT, EPIC and quanTIseq, and performing multiple downstream analyses. Tumor/normal differential expression analysis, profiling according to cancer types, patient survival analysis was performed.2. Kaplan-Meier overall survival analysis performed using the TCGA dataset for breast cancer invasive carcinoma. The statistical difference between the curves can be measured by the log-rank test. The package “survival” of the R statistical environment was used to calculate hazard ratio (HR), 95% confidence intervals (CI), and log-rank p-values.

### Statistical Analysis

Reported values are expressed as mean ± standard deviation (SD) of experiments in triplicate. Three independent biological samples have been used in each experimental set. Statistically significant differences were evaluated using the analysis of variance (two-way ANOVA) test and were considered statistically significant at the level of at least p ≤ 0.05. Statistical analysis and graphs were made using GraphPad Prism 8.2.1. software.

## Results

### *ESR2* Predicts Overall Survival Rates in Breast Cancer Patients

**Figure 1:**
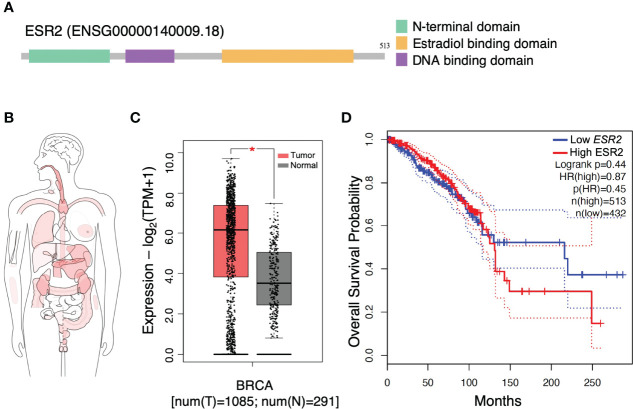
Gene structure and expression profiling of estrogen receptor beta gene (*ESR2*). **(A)** Structure of *ESR2* alternatively spliced transcript variant aberrantly expressed in breast cancer. **(B)** Interactive bodymap presenting median expression of *ESR2* in tumor samples. Scale: log_2_(TPM+1); p ≤ 0.05. **(C)** The gene expression profile across tumor samples and paired normal tissues. **(D)** *ESR2* expression is correlated to worse prognosis in breast cancer patients. GEPIA2 tool was utilized to perform these meta-analysis tests. Kaplan-Meier overall survival analysis performed using the TCGA dataset for breast cancer invasive carcinoma. The statistical difference between the curves [P value and hazard ratio (HR) value] has been calculated by the log-rank test. BRCA, breast cancer; HR, hazard ratio; TPM, transcripts per million. Asterisk (*) indicate statistically significant differences (p ≤ 0.05).

ER status is the most important discriminator of breast cancers highlighting the cardinal role of ERs as biomarkers in breast cancer progression (27). Functional studies indicate that the structural organization of main ERβ subtype suggest the ligand-induced conformational changes that explain distinct genomic and non-genomic transcriptional actions (**Figure 1A**) (3). Interactive bodymap indicates that *ESR2* is aberrantly expressed in several tumor tissues (**Figure 1B**). Regarding breast cancer, *ESR2* expression is 2-fold higher in invasive breast carcinoma as compared to normal breast tissues (**Figure 1C**). Notably, Kaplan-Meier survival analysis revealed that high levels of *ESR2* in patients with invasive breast carcinoma, demonstrate a significantly lower overall survival, as compared to breast tumors with low *ESR2* expression (**Figure 1D**). This suggests the crucial role of ERβ in prognosis of aggressive breast cancer and that its targeting may be beneficial for effective management of this malignancy.

### Expression Traits of Matrix Signaling Mediators Following *ESR2* Suppression

**Figure 2:**
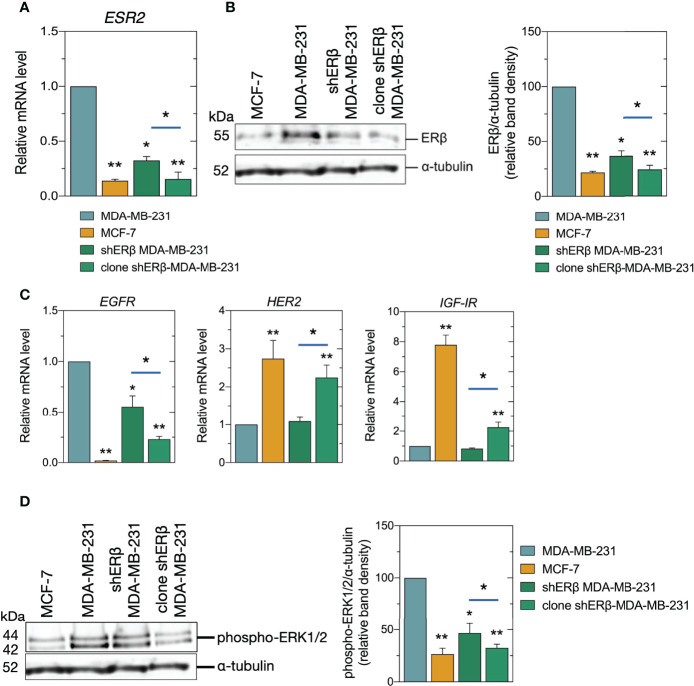
Expression of *ESR2*, ERβ protein levels, and signaling mediators in breast cancer cells. **(A)** Real-time PCR analysis of *ESR2* in MCF-7, MDA-MB-231, shERβ MDA-MB-231 and clone shERβ MDA-MB-231 cells. **(B)** Immunoblots of ERβ and α-tubulin in the four cell lines (left panel) and quantification of protein bands (right panel). **(C)** Real-time PCR analysis of *EGFR*, *HER2* and *IGF-IR*. **(D)** Immunoblots of phospho-ERK1/2 (p44/42) and α-tubulin (left panel) and quantification of protein bands (right panel). The data are presented as the mean ± SD values (n=3). Asterisks (*), (**) indicate statistically significant differences (p ≤ 0.05 and p ≤ 0.01, respectively).

Recent work deduced that the highest possible ERβ suppression should be achieved to avoid the heterogenous genotypic and phenotypic experimental observations resulting from the ERβ variants in MDA-MB-231 TNBC cells. The mature antisense sequences that have been developed to suppress *ESR2* target three sites: 3’ untranslated region (UTR), non-coding and open reading frame (ORF). These regions are common among ERβ variants. In the direction of avoiding disorientations of still present ERβ variants, we proceeded to isolation of monoclonal cell populations in shERβ MDA-MB-231 cells as to achieve higher *ESR2* suppression rates and phenotypic homogeneity, and we named the monoclonal cell cultures, clone shERβ MDA-MB-231 cells. Indeed, clone selection in shERβ MDA-MB-231cells resulted in 90% decrease in *ESR2* levels, as compared to MDA-MB-231 cells (**Figure 2A**). Notably, *ESR2* expression levels in clone shERβ MDA-MB-231 cells seem to resemble ERα-positive and ERβ-negative MCF-7 breast cancer cells (**Figure 2A**). These results have been confirmed by ERβ protein detection; not to mention the statistically significant 70% decrease in ERβ protein levels in clone shERβ MDA-MB-231 cells as compared to the heterogenous cultures of shERβ MDA-MB-231 cells (**Figure 2B**).

The ligand-independent ER actions include the phosphorylation of growth factor receptors and the subsequent activation of protein kinase signaling pathways to regulate transcription (28). Therefore, we further evaluated the expression and activity levels of major matrix signaling molecules, as growth factor receptors and mitogen-activated protein kinases (MAPK). It has been established that the most clinically aggressive subtypes of breast cancer, are also associated with *EGFR* overexpression (29, 30), while in ERα-positive breast cancer, IGF-IR is present at high levels and its action is correlated to ER status (31). In this study, we confirmed that the less aggressive breast cancer cell line, namely MCF-7, demonstrated a slight *EGFR* expression, and 7-fold *IGF-IR* and 2.5-fold *HER2* increased levels, respectively, as compared to MDA-MB-231 cells (**Figure 2C**). Most importantly, we demonstrated that clone shERβ MDA-MB-231 cells demonstrated 75% reduced *EGFR* levels, and a 2-fold increase in *HER2* and *IGF-IR* levels as compared to MDA-MB-231 cells, resembling the expression profile of epithelial cell line (**Figure 2C**). Notably, we did not note any significant alteration in *ESR1* levels in clone shERβ MDA-MB-231 cells. Finally, as shown in **Figure 2D**, the isolation of monoclonal population of shERβ MDA-MB-231 cells resulted in 65% reduction in the phosphorylated levels of ERK1/2 MAPK, as compared to MDA-MB-231 cells, that can be further connected to the less aggressive phenotype of this cell type.

### ER Status Drives Morphological Characteristics and Metastatic Potential of Breast Cancer Cells

**Figure 3:**
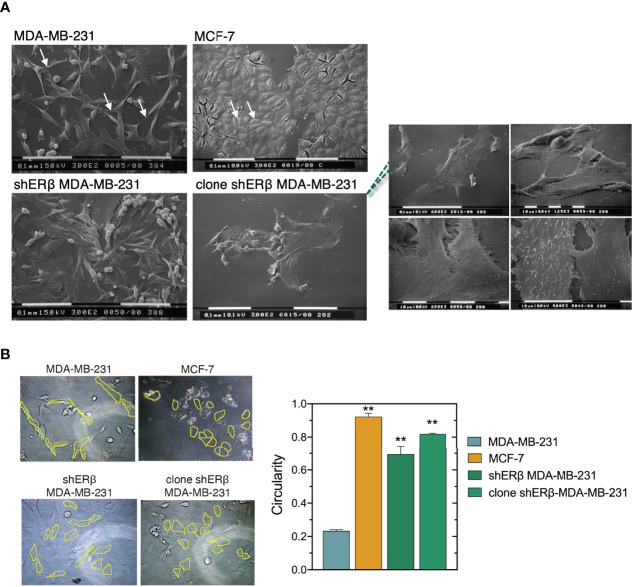
ERβ-dependent cellular morphological characteristics. **(A)** Scanning electron microscopy (SEM) of MDA-MB-231 cells shows elongated cells with long filopodia (arrows), whereas MCF-7 cultures consist of grouped cobblestone/flattened cells with tight cell junctions (arrows). ERβ-suppressed cells demonstrate phenotypic heterogeneity similar to MDA-MB-231 cells. However, the majority of ERβ-suppressed cells look like flattened with cell-cell contacts and less cytoplasmic processes. Monoclonal populations of ERβ-suppressed cells mainly contain grouped and very flattened cells with small nucleus and large cytoplasm, no microvesicles or cytoplasmic protrusions (right panel) and many cell-cell junctions. **(B)** Circularity of the different breast cancer cellular models as quantified by Image J software. The data are presented as the mean ± SD values (n=3). Asterisk, (**) indicate statistically significant differences p ≤ 0.01.

The inherent aggressive character of cancer cells dictates the initiation of metastasis, caused by an extensive matrix remodeling, loss of tissue organization and abnormal cell behavior (32). Changes in tumor microenvironment play critical roles in the migrating capability of cancer cells through the EMT program where cancer cells develop mesenchymal morphology and increased invasive capacity (33, 34). SEM analysis of MDA-MB-231 2D cultures revealed different phenotypes: mainly isolated, elongated cells with lamellipodia and filopodia protrusions, but also “cobblestone”-shaped cells and a few isolated globular-like ones (**Figure 3A**). Microvilli, microvesicles and intercellular connections were also detected on the surface of MDA-MB-231 cells, explaining the highly mobile nature of TNBC cells (35). On the other hand, the epithelial morphology of ERα-positive MCF-7 cells is pursued by the formation of cell-cell contacts, tight cell junctions and cell aggregates as confirmed by SEM analysis in 2D cultures (**Figure 3A**). Ultrastructural investigations confirmed that ERβ suppression induced the development of more round and flattened cells, significant loss of cytoplasmic protrusions and cell-cell contacts as well as the tendency to form cell aggregates. Notably, when we selected and cultured monoclonal cell populations of shERβ MDA-MB-231 cells we noticed a profound elimination of cytoplasmic protrusions and the establishment of cell cultures with flattened cells (**Figure 3A**). Notably, the high suppression rates of *ESR2* have been connected with the establishment of cultures with grouped cells exhibiting a small nucleus and large cytoplasm, no evident cytoplasmic protrusions (**Figure 3A**, right panel) and evident cell-cell contacts. Moreover, tight junctions and few microvesicles were detectable on the surface of clone shERβ MDA-MB-231 cells (**Figure 3A**, right panel).

**Figure 4:**
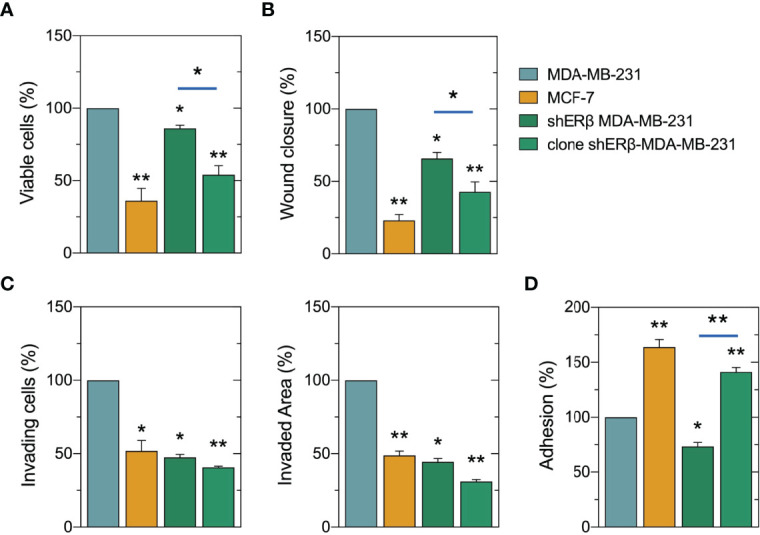
Evaluation of ERβ suppression on cell functional properties of breast cancer cells. Monoclonal populations of shERβ-MDA-MB-231 cells demonstrate much lower levels of viability **(A)**, motility **(B)** and invasiveness **(C)** and in increased adhesive capacity **(D)** as compared to shERβ MDA-MB-231 cells. The data are presented as the mean ± SD values (n=3). Asterisks (*), (**) indicate statistically significant differences (p ≤ 0.05 and p ≤ 0.01, respectively).

We further analyzed the morphological characteristics of breast cancer cells with different ER status, in respect of cell circularity (**Figure 3B**). It is well established that the architecture of tumor microenvironment constructs cancer cell characteristics, thus predicting the tumor biological behavior and invasive potential (4, 36, 37). Of note, as the value of circularity approaches 0.0, it indicates an increasingly elongated polygon; a circularity value of 1.0 indicates a perfect circle. Formation analysis in our models at first confirmed that the mesenchymal-like MDA-MB-231 cells demonstrate the lowest circularity level of 0.2. Intriguingly, the monoclonal cell populations of shERβ MDA-MB-231 cells demonstrated a 0.8 circularity rate that is much higher than that of shERβ MDA-MB-231 cells and clearly approaches MCF-7 cells’ circularity (**Figure 3B**). The above data confirm the value of ERβ in the morphology, growth and invasive properties of breast cancer cells. MDA-MB-231 breast cancer cells are characterized by increased rates of cell viability, motility and invasive capacity, followed by significant loss in cell adhesive efficiency (**Figures 4A–D**). The aggressive behavior of ERβ-positive MDA-MB-231 cells confirms their mesenchymal-like characteristics (**Figure 3A**). The striking phenotypic changes in clone shERβ MDA-MB-231 cells were guided by critical alterations in functional properties of these cells. In particular, clone shERβ MDA-MB-231 cells demonstrated harsh decrease in viability, motility and invasive capacities, along with a profound increase in adhesion capability, as compared to aggressive ERβ-positive MDA-MB-231 cells (**Figures 4A–D**). Intriguingly, these changes were much more evident than in heterogenous shERβ MDA-MB-231 cells, approaching the levels of epithelial MCF-7 cells. Collectively, these novel data suggest the prominent role of ERβ suppression in the establishment of a permanently less aggressive phenotype as depicted in the monoclonal cell populations of clone shERβ MDA-MB-231 cells.

### Reprogramming EMT and ECM Degradation in Monoclonal Cultures of shERβ MDA-MB-231 Cells

**Figure 5:**
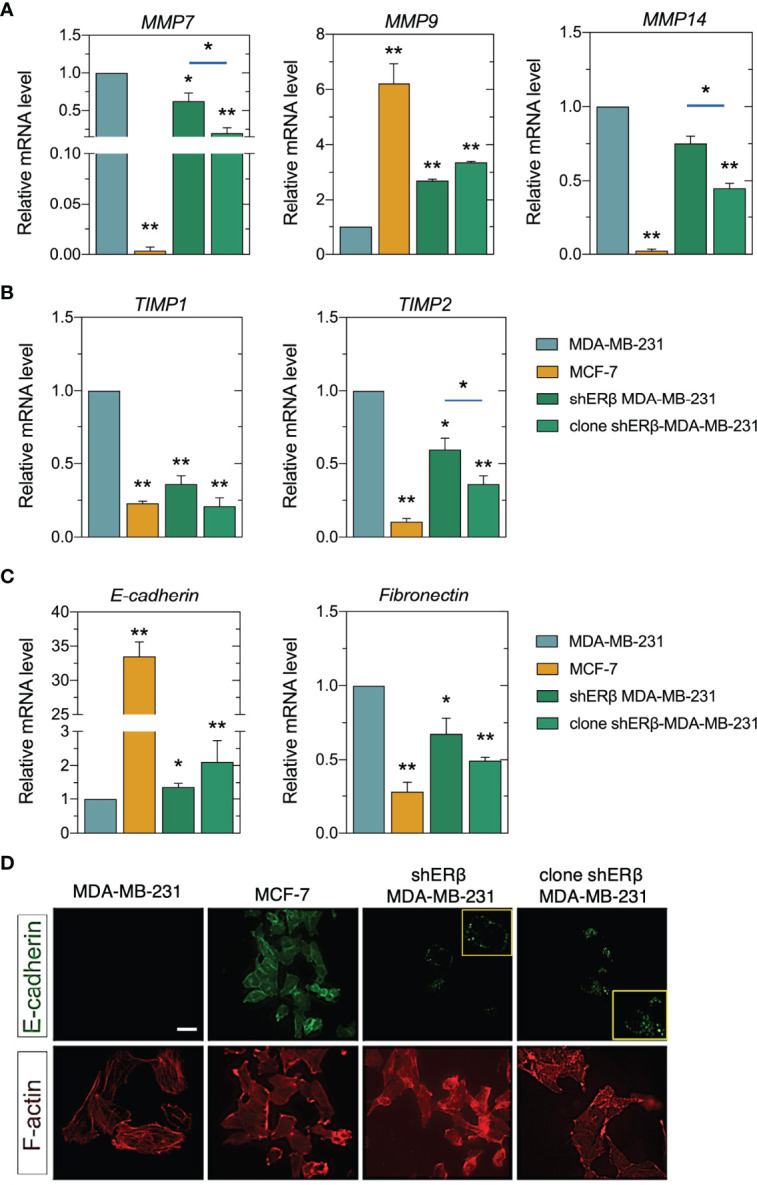
ERβ regulates matrix composition in breast cancer cells. **(A)** Real-time qPCR analysis of major MMPs (*MMP7*, *MMP9*, *MMP14*) and their endogenous inhibitors (*TIMP1*, *TIMP2*) **(B)**. **(C)** Real-time qPCR analysis of EMT biomarkers, *E-cadherin* and *fibronectin*. The mRNA levels were studied using GAPDH as reference gene. Asterisks (*), (**) indicate statistically significant differences (p ≤ 0.05 and p ≤ 0.01, respectively). **(D)** Immunofluorescence analysis of E-cadherin (green) and F-actin (red). Scale bar, 10μm.

The conversion of early-stage tumors into invasive malignancies is a hallmark in tumorigenesis and is mediated by the actions of matrix degrading enzymes, as the proteolytic MMPs, that directly mediate EMT program (7). In this study, we revealed that *ESR2* high suppression rates in monoclonal shERβ MDA-MB-231 cells decreased the mRNA levels of MMP7 and MMP14, 70% and 55%, respectively, as compared to the highly invasive MDA-MB-231 cells (**Figure 5A**), whereas it led to a 3-fold increase in MMP9 levels (**Figure 5A**), an epithelial-derived MMP that acts as tumor suppressor in many types of cancer (38, 39). These changes in clone shERβ MDA-MB-231 cells co-existed with *TIMP1* and *TIMP2* decrease at 75% and 60%, respectively as compared to MDA-MB-231 cells (**Figure 5B**). Our data pinpointed that the expression profile of clone shERβ MDA-MB-231 cells in respect of *MMPs* and *TIMPs* resembles that of low metastatic MCF-7 breast cancer cells (**Figure 5A**, **B**). Furthermore, as shown in **Figures 5C, D**, the 90% *ESR2* suppression in monoclonal shERβ MDA-MB-231 cells resulted in a 2.5-fold increase in E-cadherin mRNA and protein levels, the major protein in adherens junctions, serving as an epithelial marker (40). Immunofluorescence analysis revealed the characteristic E-cadherin expression dots, in the monoclonal populations of shERβ MDA-MB-231 cells. These cells express this glycoprotein in cell junctions, as compared to MDA-MB-231 cells where E-cadherin staining is negative (**Figure 5D**, yellow frames). Notably, E-cadherin protein expression is more evident in clone shERβ MDA-MB-231 cells than in heterogenous shERβ MDA-MB-231 cells (**Figure 5D**). These results confirm the adhesive profile of clone shERβ MDA-MB-231 cells as depicted in **Figure 4D**. E-cadherin protein expression in MCF-7 epithelial cells with tight junctions confirmed the existence of this cell adhesion molecule in cell junctions (**Figure 5D**). *ESR2* depletion resulted also in a 50% reduction in *fibronectin* levels, as compared to MDA-MB-231 cells (**Figure 5C**). Fibronectin that promotes EMT and serves as a scaffold for fibrillar ECM (41), explaining its high mRNA levels in aggressive ERβ-positive MDA-MB-231 cells compared to the 70% decrease in MCF-7 epithelial cells (**Figure 5C**). This screening highlights the ERβ-mediated switch of the mesenchymal-to-epithelial transition (MET) trait in homogenous monoclonal cultures of shERβ MDA-MB-23 cells.

The invasive capacity of breast carcinoma cells is broadly connected to their phenotype, which determines EMT, cell-matrix and cancer cell-stroma interactions, critical to initiate a premetastatic niche (42). Striking alterations in morphological characteristics, lamellipodia deletion (**Figure 3A**), E-cadherin increment along with *fibronectin* loss, has led to robust cytoskeleton rearrangement in clone shERβ MDA-MB-231 cells (**Figure 5D**). F-actin staining for cytoskeleton revealed a clearly condensed cytoskeleton network in these cells, resembling F-actin microtubule network of MCF-7 cells, as compared to the characteristic mesenchymal-like cytoskeleton of MDA-MB-231 cells. All things considered, these findings highlight that *ESR2* could play a role in matrix remodeling, hence the expression of certain MMPs (i.e., MMP7, MMP9, MMP14) are critical factors for the degradation and reorganization of ECM components. *ESR2* also drives cytoskeletal rearrangement, and the expression profiles of major EMT markers.

### ERβ Maximum Suppression Prevents *In Vivo* ERβ-Evoked Tumorigenesis

**Figure 6:**
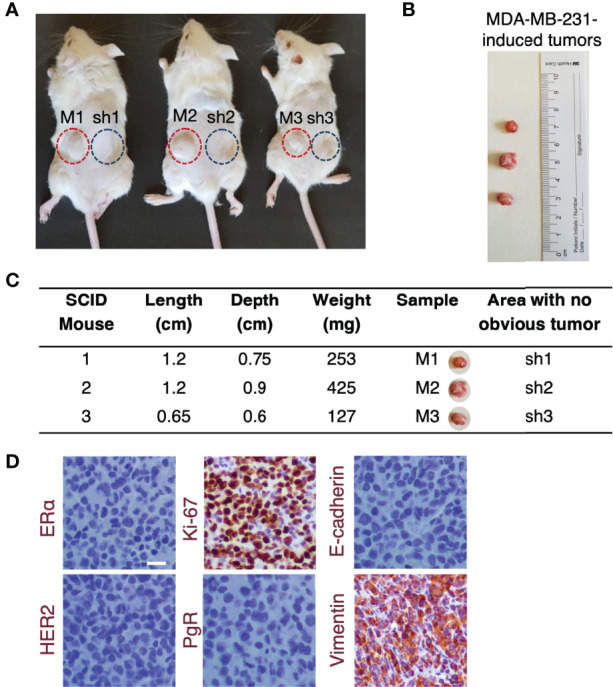
The highest *ESR2* suppression in MDA-MB-231 cells prevents tumorigenesis *in vivo*. **(A)** 10^6^ MDA-MB-231 (red cycles) and clone shERβ MDA-MB-231 (blue cycles) cells were inoculated in two sites in the back of three female SCID mice. Tumor formation was observed only in sites where MDA-MB-231 cells were injected (M1, M2, M3) and no obvious tumors were detected following injections with clone shERβ MDA-MB-231 cells (sh1, sh2, sh3). **(B)** Size of the three tumors extracted from mice following injections with MDA-MB-231 cells (M1, M2, M3) for four weeks and **(C)** *ex vivo* tumor measurements. **(D)** Immunohistochemical analysis of specific breast cancer markers (i.e., ERα, PgR, HER2, Ki-67, E-cadherin, vimentin) in the MDA-MB-231 tumor tissue confirms the mesenchymal phenotype of tumor cells. Positive staining is indicated with brown. Scale bar, 10μm.

On account of the intrigued role of ERβ in mediating MET process by altering cellular characteristics and functions, F-actin cytoskeleton rearrangement and ECM reorganization, the *in vivo* tumorigenic capacity of this ER was assessed. 10^6^ MDA-MB-231 and clone shERβ MDA-MB-231 cells were injected in two sites in the back of three female SCID mice and tumor formation was monitored for four weeks (**Figure 6A**). Intriguingly, after four weeks, *in vivo* tumor formation was observed exclusively in sites where MDA-MB-231 cells were injected (**Figure 6A**). The size and expression profile of MDA-MB-231-formed tumors was calculated with *ex vivo* size measurements (**Figures 6B, C**) and immunochistochemistry analysis of major TNBC markers, including ERα, PgR, HER2, Ki-67, E-cadherin and vimentin (**Figure 6D**). Specifically, MDA-MB-231-generated tumors express vimentin and Ki-67, whereas the loss of ERα, PgR, HER2 and E-cadherin confirmed the aggressive phenotype of MDA-MB-231 cells and the necessity of targeting the ER that mediates this behavior, namely ERβ. In light of these facts, we conclude that ERβ directly controls *in vivo* tumorigenesis and the aggressive profile of TNBC cells, as the capacity of MDA-MB-231 cells to form tumors is vanished following *ESR2* practically total suppression, highlighting the importance of its molecular targeting.

## Discussion

**Figure 7:**
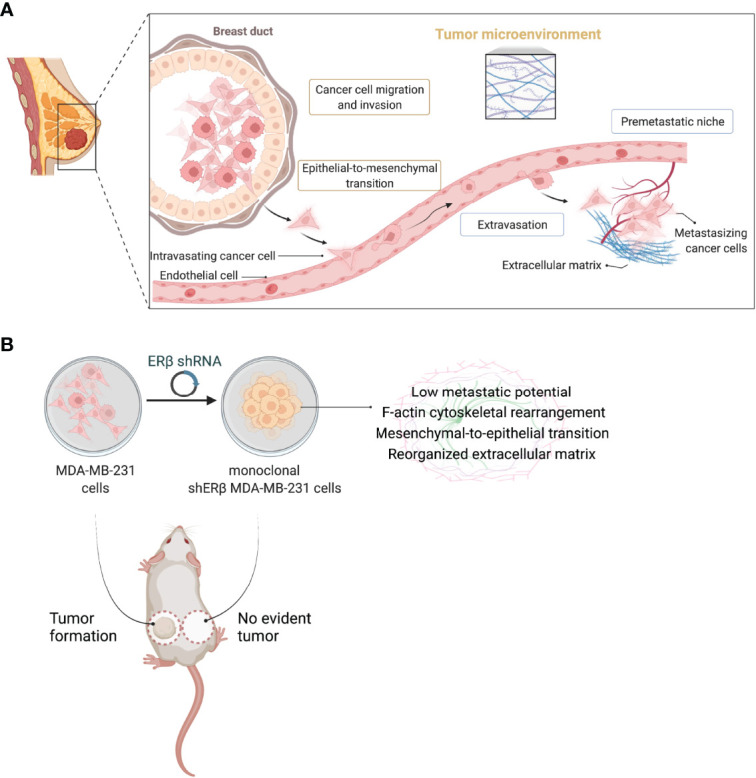
ERβ globally mediates the behavior of triple-negative breast cancer cells. **(A)** Schematic depiction of major steps during breast cancer metastasis from a primary breast tumor of mesenchymal-like cancer cells, as the ERβ-positive triple-negative breast cancer (TNBC) cells. ERβ-dependent epithelial-to-mesenchymal transition alters the behavior of TNBC cells to advance high dynamics to invade endothelial barrier and enter blood stream. Intravasated cells follow the opposite process in order to be transferred through the blood stream. The extravasation of metastatic cancer cells creates a favourable microenvironment for premetastatic niche formation in secondary tissues and distant organs that is characterized by extensive matrix remodelling and the activation of the angiogenic switch. **(B)** Schematic representation delineating the cardinal role of ERβ in modulating the invasive phenotype of MDA-MB-231 TNBC cells. ERβ suppression and isolation of monoclonal populations transforms MDA-MB-231 cells into a less aggressive subtype that prevents *in vivo* tumorigenesis. Please consult the manuscript for additional details. Created with BioRender.com.

The complex structure of solid tumors together with the interactions of cancer cells with the surrounding stroma and matrix components stimulate the initiation of a premetastatic niche that promotes metastasis to distant sites (8, 43–45) (**Figure 7A**). Alterations in the expression profiles of ECM structural components, including among others, PGs, hyaluronan, growth factor receptors, MMPs, and signaling stimulators, foresee the variations in cancer cell behavior, and construct the homes for metastasis (7, 46–48). Even though the significance of ERβ is less clarified in breast cancer progression than its isoform, ERα, probably due to the existence of several alternatively spliced ERβ variants; however, the potential of ERβ targeting in aggressive breast cancer subtypes, as TNBC, has gained attention over the years (49). In this study, we reported that high *ESR2* expression rates have been correlated to decreased overall survival rates in breast cancer patients diagnosed with invasive breast carcinoma. Recent reports indicate that ERβ target genes mostly regulate cell survival, movement, and growth (50), and that ERβ signaling pathway intersects with EGFR cascade to mediate TNBC cell morphology and stemness (13, 15). These data have been corroborated by the strong implication of ERβ in EMT process since it acts as EMT promoter by activating the TGFβ/Smad3 pathway to promote tumor growth and invasion in metastatic renal cell carcinoma (51). We demonstrated that ERβ maximum suppression (90%) leaded to transformed MDA-MB-231 clones that slightly express *EGFR*, whereas the expression rates of *IGF-IR* and *HER2* have been induced. Notably, IGF-IR signaling is reported to drastically lower the aggressive potential of breast cancer cells (31). The intracellular signaling pathway of EGFR receptor tyrosine kinase, includes the activation of Ras/MAPKs and PI3K/AKT and is involved in various aspects of breast cancer cell growth (52). Here we report that the slight *EGFR* expression is correlated with a robust decrease in ERK1/2 phosphorylation in monoclonal shERβ MDA-MB-231 cells, compared to the excessive phosphorylation in the invasive MDA-MB-231 cells. In previous studies, we have demonstrated that the distinct ER status of breast cancer cells is correlated to different microRNA (miRNA) epigenetic signatures and that specific miRNAs (i.e., miR-10b, miR-145 and miR-200b) are possible biomarkers for regulating breast cancer cell behavior by interacting with matrix mediators (19, 20, 53, 54). Especially for ERβ, this ER is the epigenetic mediator of miR-10b, miR-145 and miR-92, specific miRNAs implicated in breast cancer progression (19, 55). These findings clearly indicate the potential of ERβ-targeting in aggressive breast cancer, however little is known about its implication in tumor formation *in vivo*.

Enzymatic proteolysis is critical for matrix functionality and integrity, tissue homeostasis and cell signaling. Matrix degradation is predominantly orchestrated by bioactive MMPs that are not only responsible for ECM remodeling, but they also control the activities of other matrix components, suggesting their fundamental role in complex biological processes during cancer progression (7, 56). Moreover, it is well established that extensive or even dysregulated matrix remodeling generate molecular cues to promote tumorigenic processes. In datasets of primary breast tumors, high expression levels of a subset of MMPs, including MMP7 and MMP14, are correlated to poor prognosis and decreased survival rates (7, 57, 58). However, MMP9 expression varies among the molecular subtypes of breast carcinoma (39, 59). Clearly, ERβ has a critical role in reducing the expression levels of MMPs, *MMP7* and *MMP14*, and their endogenous inhibitors, *TIMP1* and *TIMP2*, underscoring the notion that ERβ-evoked MMPs elevated levels in TNBC cells is cardinal for the initiation of the premetastatic niche in these cells. Intriguingly, monoclonal populations of shERβ MDA-MB-231 cells demonstrate a strong increase in *MMP9* levels following the profile of the MCF-7 breast cancer cells of low metastatic potential. This suggests that TNBC has a stronger clinical value in predicting metastasis rather than any of the other biological factors examined.

The formation of cell junctions is controlled by interactions of the transmembrane glycoproteins, mainly E-cadherin and intracellular components as β-catenin. The multifunctional complex of cell junctions includes the organization of actin cytoskeleton and the stabilization of cell-cell adhesion (60, 61). ERβ has an active role in regulating major EMT modulators, as its absence in monoclonal populations of shERβ MDA-MB-231 cells clearly boosted E-cadherin protein expression in the newly formed cell-cell junctions that these cells form. In respect to this observation, *fibronectin*, a fibrillar protein regulating cell-matrix adhesion and fibro-proliferative condition in diseased tissues (62), is diminished compared to the aggressive MDA-MB-231 TNBC cells, depicting that the ERβ-dependent mesenchymal characteristics connected to EMT initiation have been lost. Lamelipodia and long filopodia dynamics consist the cell motor pool mediating cell adhesion, motility, EMT and invasive capacity of breast cancer cells (63–65). Notably, ERβ-suppression in monoclonal cell populations of shERβ MDA-MB-231 cells clearly transformed TNBC cells to those with a less aggressive phenotype as explained by the fact that these cells lost the invasive morphology of MDA-MB-231 cells that is explained by the spindle-like shape and the long filopodia and lamellipodia cellular protrusions. Intriguingly, clone shERβ MDA-MB-231 cells gained the acquired morphological characteristics of MCF-7 epithelial-like cells, implying the flattened shape, loss of cellular protrusions and the formation of cell aggregates (**Figure 7B**). This transformation in clone shERβ MDA-MB-231 cells resulted in robust F-actin cytoskeletal rearrangement and the establishment of condensed F-actin network that is related to the decreased viability, motility and invasion to collagen type I of these cells (66). In addition, previous studies suggest that actin dynamics directs the suppression of cell invasion in HER2-positive and ERα-positive tumors (67). Together, these findings suggest that ERβ is required for the establishment of actin structures and cellular characteristics during TNBC progression.

Collectively, the main goal of this study focused on unravelling the effects of highest possible ERβ suppression in ERβ-positive MDA-MB-231 TNBC cells on matrix composition, EMT program and *in vivo* tumor growth. The pioneering discovery of this study summarized that ERβ serves as one of the major biomolecules in this aggressive subtype of breast carcinoma and that its suppression is capable of leading to the total prevention of tumorigenesis (**Figure 7**). The molecular axis enclosing ERβ, matrix effectors (i.e., cell receptors and MMPs), and principal EMT mediators (i.e., E-cadherin and fibronectin), is critical for breast cancer progression and definitely affects response to endocrine therapy.

The apparent advantage of precise ERβ inhibition in guiding a paradigmatic shift to a less aggressive cell population with no apparent dynamics in forming tumors *in vivo* may be of great clinical interest opening new horizons in research of personalized therapeutics for TNBC. Exploiting the pharmacological targeting of ERβ as a powerful biomarker in TNBC may be among the bio-tools for improving the management and survival rates of breast cancer patients.

## Data Availability Statement

The raw data supporting the conclusions of this article will be made available by the authors, without undue reservation.

## Ethics Statement

The animal study was reviewed and approved by N.C.S.R "Demokritos" institutional guidelines conforming to international standards and the protocols were approved by the relevant committee of the Veterinary Direction, Greek Ministry of Rural Development and Food (Permission No. 193900).

## Author Contributions

ZP and NK contributed to the conception and design of the work; ZP, MF, AP, DK, and NK involved in methodology; ZP, AK, MF, and VZ performed formal analysis, acquisition, and data interpretation; ZP prepared the original draft and figures; NK substantively revised draft and supervised the study. All authors have read, reviewed and agreed to the published version of the manuscript.

## Funding

ZP acknowledges funding by the Horizon 2020 project NMBP-TO-IND-2018-2020 (MIS953152), and NKK by the Action for the Strategic Development on the Research and Technological Sector (MIS5033644), funded by the Operational Programme ‘Competitiveness, Entrepreneurship and Innovation’ (NSRF 2014-2020), and co-financed by Greece and the European Union (European Regional Development Fund).

## Conflict of Interest

The authors declare that the research was conducted in the absence of any commercial or financial relationships that could be construed as a potential conflict of interest.

## Publisher’s Note

All claims expressed in this article are solely those of the authors and do not necessarily represent those of their affiliated organizations, or those of the publisher, the editors and the reviewers. Any product that may be evaluated in this article, or claim that may be made by its manufacturer, is not guaranteed or endorsed by the publisher.
