# Supplementary material for: ESR2 Drives Mesenchymal-to-Epithelial Transition in Triple-Negative Breast Cancer and Tumorigenesis In Vivo
Source: Front Oncol. 2022 Jun 3;12:917633. doi: 10.3389/fonc.2022.917633 (PMC9203970; doi:10.3389/fonc.2022.917633)
Supplement: Supplementary file 1 [file Table_1.docx]

***SUPPLEMENTARY MATERIAL***

**Table 1.** List of real-time PCR primers used in this study.

| **Gene of interest** | **Primer Sequence (5’-3’)** | | **Annealing temperatureT_annealing_ (°C)** |
| --- | --- | --- | --- |
| *ERβ* | F | TCC ATG CGC CTG GCT AAC | 60 |
|  | R | CAG ATG TTC CAT GCC CTT GTT A |  |
| *EGFR* | F | ATG CTC TAC AAC CCC ACC AC | 60 |
|  | R | GCC CTT CGC ACT TCT TAC AC |  |
| *HER2* | F | CTG CAC CCA CTC CTG TGT GCA CCT G | 60 |
|  | R | CTG CCG TCG CTT GAT GAG GAT C |  |
| *IGF-IR* | F | ACG AGT GGA GAA ATC TGC GG | 60 |
|  | R | ATG TGG AGG TAG CCC TCG AT |  |
| *E-Cadherin* | F | TAC GCC TGG GAC TCC ACC TA | 60 |
|  | R | CCA GAA ACG GAG GCC TGA T |  |
| *Fibronectin* | F | CAT CGA GCG GAT CTG GCC C | 60 |
|  | R | GCA GCT GAC TCC GTT GCC CA |  |
| *MMP7* | F | GCT GGC TCA TGC CTT TGC | 60 |
|  | R | TCC TCA TCG AAG TGA GCA TCT C |  |
| *MMP9* | F | TTC CAG TAC CGA GAG AAA GCC TAT | 60 |
|  | R | GGT CAC GTA GCC CAC TTG GT |  |
| *MMP14* | F | CAT GGG CAG CGA TGA AGT CT | 60 |
|  | R | CCA GTA TTT GTT CCC CTT GTA GAA GTA |  |
| *TIMP-1* | F | CGC TGA CAT CCG GTT CGT | 60 |
|  | R | TGT GGA AGT ATC CGC AGA CAC T |  |
| *TIMP-2* | F | GGG CAC CAG GCC AAG TT | 60 |
|  | R | CGC ACA GGA GCC ATC ACT |  |
| *GAPDH* | F | AGG CTG TTG TCA TAC TTC TCA T | 60 |
|  | R | GGA GTC CAC TGG CGT CTT |  |
